# Risk Assessment of Heavy Metal Pollution in Farmland Soils at the Northern Foot of the Qinling Mountains, China

**DOI:** 10.3390/ijerph192214962

**Published:** 2022-11-14

**Authors:** Aidi Huo, Xing Wang, Zhixin Zhao, Luying Yang, Fangqian Zhong, Chunli Zheng, Ningbo Gao

**Affiliations:** 1 Key Laboratory of Subsurface Hydrology and Ecological Effects in Arid Region, Ministry Education, Chang’an University, Xi’an 710054, China; 2 School of Water and Environment, Chang’an University, Xi’an 710054, China; 3 Xi’an Monitoring, Modelling and Early Warning of Watershed Spatial Hydrology International Science and Technology Cooperation Base, Chang’an University, Xi’an 710054, China; 4 Department of Environmental Sciences and Engineering, Xi’an Jiaotong University, Xi’an 710049, China; 5 Xi’an International Joint Research Center on Solid Waste Recycling and Utilization, Xi’an Key Laboratory of Solid Waste Recycling and Resource Recovery, School of Energy and Power Engineering, Xi’an Jiaotong University, Xi’an 710049, China

**Keywords:** soil pollution, heavy metal, geological cumulative index, pollution assessment

## Abstract

To provide scientific basis for the prevention and control of heavy metal pollution, a field investigation, sample collection and analysis of the heavy metal content in farmland soils at the northern foot of the Qinling Mountains were conducted. Based on the comparative analysis of the single pollution index method, the Nemerow comprehensive pollution index method, the geological accumulation index method, the potential ecological hazard index method, and the geological accumulation index method were used to comprehensively analyze and evaluate the risk of soil heavy metal pollution. The results showed that the heavy metal pollution of farmland soil at the northern foot of the Qinling Mountains was severe, among which Hg and Cr pollution was relatively obvious. Taking the soil screening values of agricultural land as the standard, the quantity of element Hg in agricultural soils at the northern foot of the Qinling Mountains was higher than the relevant screening value. In the two sample sites investigated, the intensity of the heavy metal accumulation index in Baoqizhai Village was Hg > Cr > Cu > As > Pb, and in Dayangyu Village it was Cr > Cu > As > Pb. Among them, in Baoqizhai Village it shows the heavy pollution caused by Hg ($I_{geo}=$ 3.42) and the light pollution caused by Cr ($I_{geo}$ < 1) in the two areas. Hg is mostly affected by mining activities and its atmospheric subsidence. At the same time, Cr is mainly derived from the weathering of rock parent material and is also affected by anthropogenic factors to a certain extent. The accumulation of heavy metals in the farmland soil around the northern foot of the Qinling Mountains was relatively high, posing a threat to the surrounding soil environment. Therefore, it is urgent to control farmland soil environmental pollution.

## 1. Introduction

With the development of industry and agriculture, various heavy metals and organic chemicals are released into the atmosphere, hydrosphere, pedosphere, and biosphere. Heavy metals can be accumulated in organisms and consequently enriched through the food chain. Organic chemicals and heavy metal pollution can, thus, cause severe health problems in the population. The northern foot of the Qinling Mountains generally refers to the area from the north of the main ridge of the Qinling Mountains to the south of the Weihe River, which is the transition zone between the lofty mountains and the vast Guanzhong Plain. This geographical particularity makes it pivotal in protecting the Qinling Mountains [1]. The shallow mountainous area at the northern foot of the Qinling Mountains is the transitional zone between the mountain and the plain, that is, the ecotone zone between the mountain ecosystem and its adjacent plain ecosystem. The ecosystem is fragile and transitional [2]. Due to the relatively flat terrain in this area, villages are widely and densely distributed, which are greatly affected by human beings. The original natural vegetation and soil are seriously damaged, the area of barren hills and slopes is increasing yearly, and the secondary forests are distributed in disorder, which also aggravates the problem of heavy metal pollution in soil [3]. The Qinling Mountains are the boundary between the north and the south of China’s climate and represent a significant ecological security barrier. They are China’s ancestor vein and gene pool, the critical water conservation area of the Yellow River and Yangtze River basin, and China’s “central water tower”. Protecting the ecological environment of the Qinling Mountains, especially the northern foot of the Qinling Mountains, is of great and far-reaching significance to ensure the long-term prosperity and sustainable development of the Guanzhong Basin, and even the Chinese nation.

As the traditional industrial economy and agricultural economic development model ignore environmental protection, it not only induces the occurrence of geological disasters [4,5], but also leads to relatively heavy air, water, and soil pollution [6]. Water and air pollution are easy to find, and related technologies are adopted in time for effective governance. Soil pollution has solid concealment and mobility characteristics; the investigation and management are relatively complex. In particular, heavy metals have a strong destructive effect on the basic structure of soil and the ecological environment, which not only seriously restricts the robust growth of vegetables, fruits, and grains, but also easily leads to excessive harmful substances in agricultural products, affecting people’s physical and mental health. The northern foot of Qinling Mountains in Shaanxi is mainly located south of Xi’an, with six administrative districts and counties and one development zone (Zhouzhi County, Huyi District, Chang’an District, Baqiao District, Lantian County, Lintong District, and Hi-tech Zone) in Xi ‘an. The region’s total area is 6466.93 km^2^, accounting for 62.27% of the total area of the northern foot of the Qinling Mountains. It is the most biodiversity-rich region in the north foot of the Qinling Mountains. In it are located the first national botanical garden in China, Qinling National Botanical Garden, and the first national park, Giant Panda National Park; the number of vertebrates in the region accounts for 70% of the Qinling Mountains. Since 1999, Shaanxi Province has implemented a beautiful mountain and river project, with soil and water conservation and returning farmland to the forest (grass) as the main subjects in the whole province. However, after nearly 20 years of restoration, the ecological environment in the shallow mountainous area at the northern foot of the Qinling Mountains, where villages are widely spread, has not been restored. There are even excessive development and illegal construction, leading to the continuous deterioration of the ecological environment, the destruction of natural vegetation, the pollution of heavy metals in the soil, and other problems that have caused irreversible damage to the original ecological environment. It is highly urgent to strengthen the soil environment control at the northern foot of the Qinling Mountains, implement the project of beautiful mountains and rivers, protect biological resources, and improve the ecological environment.

To protect the ecological environment in the northern foot of the Qinling Mountains, Shaanxi Province has issued “Ecological Protection Regulations of Qinling Mountains in Shaanxi Province” and an “Ecological Environment Protection Rectification Plan in Qinling Mountains of Shaanxi Province”. It is banning all illegal development and construction activities in shallow mountainous areas and restoring the damaged land promptly. Based on this opportunity, this study took the lead in analyzing soil heavy metal pollution in the regreening farmland after demolishing illegal construction at the northern foot of the Qinling Mountains. The land in the Yangyu watershed of Jiaodai Town in the Lantian section of the shallow mountainous area at the northern foot of the Qinling Mountains is the research object. The content and distribution characteristics of heavy metals in the soil of regreening farmland were investigated. The pollution assessment of heavy metals in farmland soil was conducted using the geo-accumulation index method to explore the pollution sources of heavy metals in this area. This provides data support for finding out the soil pollution situation in this area and has vital practical significance for the subsequent improvement of soil environmental quality, the removal of heavy metal pollution, the timely regreening of human disturbance land, and other ecological environment protection construction work in this area. It can also provide a scientific theoretical basis for the restoration and conservation of vegetation and the overall improvement of natural environment service function in this area.

## 2. Materials and Methods

### 2.1. Study Area

**Figure 1:**
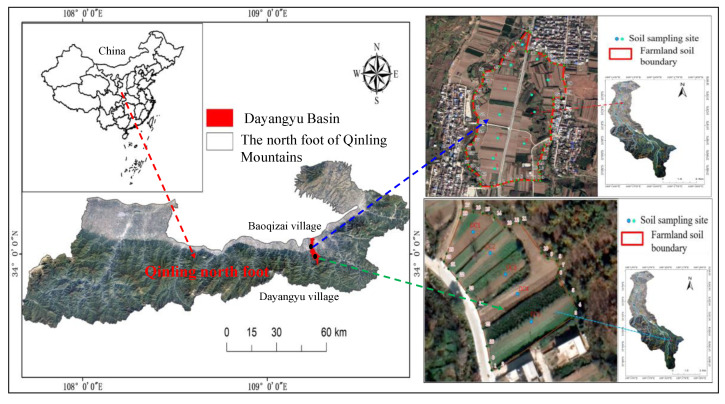
Geographical location diagram of the study area.

The research area is located in the administrative villages under the jurisdiction of Jiaodai Town, Lantian County, Xi’an City, Shaanxi Province—Baoqizhai Village and Dayangyu Village (33.971°–34.098° N, 109.183°–109.304° E); the specific location is shown in Figure 1. During the Ming and Qing Dynasties and the Republic of China, it was Jiaodai Town. At the beginning of the founding of the People’s Republic of China, it was a township. In 1958, a commune was established, and it changed to a town in 1984. It is located in the southern part of the county, at the northern foot of the Qinling Mountains. It covers an area of 45 km^2^ and has a population of 23,000. Tangyu Road, Jiaodai to Xiaozhai Road crisscross the territory. It is a warm tropical semi-humid continental monsoon climate zone, and the four seasons are clear between cold and warm, dry and wet. The average annual frost-free period is 219 days, yearly precipitation is 879 mm, the annual temperature is 13 °C, and zonal soil is yellow-brown. Shallow mountainous areas are primarily low mountains with a relative height of 500 m or below. As the slope is relatively gentle and there is a certain amount of loess deposition, most are mountainous agricultural land. Due to frequent human activities, the original natural vegetation is seriously damaged. Most of the existing vegetation is an artificially planted secondary forest, including a part of farmland returned to woodland, and the vegetation type is mixed broadleaf–conifer forest. Plot 1 is located east of Baoqizhai Village and north of Renjiayuan Village in Yangyu Valley, Jiaodai Town, covering an area of 290,861.682 m^2^. The center coordinate of the plot is 109.239° E, 34.044° N. The east and west sides of the plot are Baoqizhai Village, the south side is Renjiayuan Village, the east and west sides are Yangyugou and a pond, and the north side is the intersection of the ditches. Plot 2 is located northwest of Dayangyu Village, Jiaodai Town, Lantian County, Xi’an City, Shaanxi Province, covering an area of 5275.43 m^2^. The center coordinate of the plot is 109.2681° E, 33.9979° N. The plot’s east side is mountainous, the south side is Dayangyu village, the west side is Dayangyu ditch and pond, and the north side is farmland.

### 2.2. Sample Collection and Analysis Methods

#### 2.2.1. Soil Sampling and Structure

According to the national standard “the Technical Specification for soil Environmental monitoring” (HJ/T 166-2004), farmland soil in the study area was collected in August 2022, and a total of 14 samples of topsoil (0–20 cm) were collected by random distribution. The sampled farmland was covered with loess, mainly planting corn, pepper, tomato, eggplant, and other crops. The collected sample was placed into a polyethylene sample bag, the location of the sampling point was recorded, and a label was stuck. The collected soil samples were air-dried at room temperature, ground with a mortar, and passed through 830 and 150 m sieves, respectively.

**Figure 2:**
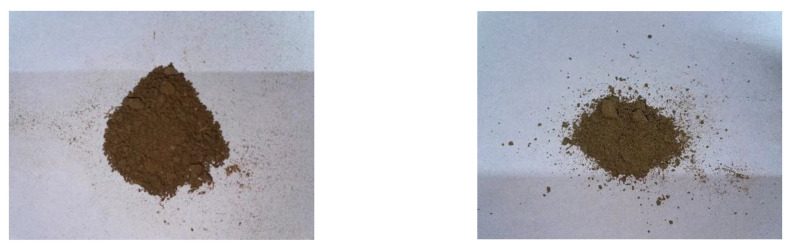
Macro photo of soil in Baoqizhai Village (**left**). Macro photo of soil in Dayangyu Valley (**right**).

Figure 2 shows the macro photo of the soil sample in Baoqizhai Village. Overall, the soil structure was relatively loose, with good porosity, ventilation, moisture absorption, and water retention ability. As a result, the soil color of Baoqizhai Village was dark and reddish brown. In contrast, the soil color of Yangyangyu Village was light and yellowish brown, which may be caused by the difference in soil moisture content and element content between the two places. In addition, in terms of soil structure, soil samples from both places have large, hard, and dense massive structures and small, loose, and porous granular structures that are nearly spherical.

#### 2.2.2. Extraction of Heavy Metals from The Soil

The chemical components of soil samples in Baoqizhai were analyzed by a handheld X-ray fluorescence spectrometer. Heavy metals were extracted from the soil by microwave digestion. First, 0.1 g of the dried and ground soil sample was placed into a polytetrafluoroethylene (PTFE) digestion tank. Then, 6 mL HNO_3_, 2 mL HCl, and 2 mL HF were added in turn. To ensure complete digestion, the samples were predigested at 70 °C for 30 min on an electric heating plate. Afterward, the samples were digested in a microwave digester (Multiwave Pro, Anton Paar, Vienna, Austria) at 210 °C for 45 min. After digestion, 2 mL perchloric acid was added to the PTFE digestion tank at room temperature until no residue appeared. Then the PTFE digestion tank was heated on an electric heating plate for 90 min at 180 °C. The remaining solution was transferred to a 50 mL volumetric flask. The volumetric flask was diluted to 50 mL with 2% HNO_3_. The diluted digestion solution was passed through a 0.45 μm filtration membrane to be tested.

#### 2.2.3. Determination of Heavy Metals in Soil

The contents of heavy metals in the soil were measured by an inductively coupled plasma emission spectrometer (ICP-AES, Shimadzu ICPE-9000, Sydney, Australia). The instrument conditions were as follows. Wavelength range: 167–800 nm; optical system: echelle splitter; elements in the vacuum ultraviolet region correspond to vacuum splitter; splitter temperature: thermostatic control; detector: semiconductor detector CCD, 1 million pixels; RF radiofrequency generator: crystal oscillation type; frequency: 27.12 MHz.

### 2.3. Evaluation Method of Heavy Metal Pollution Characteristics

#### 2.3.1. Evaluation Criterion

This study selected the risk screening value of the “Soil Environmental Quality Risk Control Standard for Soil Contamination of Agricultural Land” (GB15618-2018) as the reference value. When the soil in the sample area is higher than the risk screening value specified in Table 1, the risk of soil pollution in agricultural land may exist, and soil environmental monitoring and collaborative monitoring of agricultural products should be strengthened. Therefore, the risk screening values of heavy metals are shown in Table 1.

#### 2.3.2. Single Factor Pollution Index Method

The single-factor index evaluation method is used to evaluate the pollution degree of a single pollution factor in soil. The smaller the pollution index, the lighter the pollution degree. The equation is as follows:

(1) $$P_{i}=C_{i}/S_{i}$$

where $P_{i}$ is the single factor pollution index of factor *i*, $C_{i}$ is the measured content of factor *i*, and $S_{i}$ is the standard evaluation value of factor *i*. This study used the soil pollution risk screening value in the “Soil Environmental Quality Risk Control Standard for Soil Contamination of Agricultural Land” (GB15618-2018).

#### 2.3.3. Comprehensive Pollution Index Method

The comprehensive pollution index can be used to evaluate the study area to obtain the pollution contribution degree of a certain soil heavy metal and the overall ecological risk status of the area. The calculation method is shown in Equation (2):

(2) $$P_{N}=\sqrt{\frac{{\left(\frac{C_{i}}{S_{i}}\right)}_{max}^{2}+{\left(\frac{C_{i}}{S_{i}}\right)}_{ave}^{2}}{2}}$$

where $P_{N}$ is the comprehensive pollution index, ${\left(C_{i}/S_{i}\right)}_{max}$ is the maximum pollution index of each pollutant, and ${\left(C_{i}/S_{i}\right)}_{ave}$ is the arithmetic average of the pollution index of each pollutant.

#### 2.3.4. Geo-Accumulation Index Method

The geo-accumulation index method was proposed by Muller [8], a German scientist, to evaluate the pollution degree of a single heavy metal. This method fully considers the impact of natural geological processes and comprehensive human activities on the sedimentary environment. Therefore, it can be used as a quantitative index to study the potential impact of heavy metals in soil. The equation is as follows:

(3) $$I_{geo}=log_{2}\frac{C_{i}}{k\times B_{i}}$$

where $I_{geo}$ is the geo-accumulation index, the specific classification is shown in Table 2. $C_{i}$ is the measured content of element i in the sample, and $B_{i}$ is the geochemical background value of element *i* in the sample. The soil background value in Shaanxi Province was selected in this study [9], as shown in Table 3, and k is the empirical coefficient, generally taken as 1.5.

#### 2.3.5. Potential Ecological Risk Index Method

The potential ecological risk index method contains two concepts: the potential single factor ecological risk index and the potential comprehensive ecological risk index. The equation is as follows:

(4) $$E_{i}=T_{r}^{i}\frac{C_{f}^{i}}{C_{n}^{i}}$$

(5) $$RI=\overset{m}{\underset{i=1}{\sum }}E_{i}$$

where $E_{i}$ is the single factor potential ecological risk index, $T_{r}^{i}$ is the toxicity coefficient of heavy metal *i*, $C_{f}^{i}$ is the measured content of heavy metal *i*, $C_{n}^{i}$ is the background reference value of heavy metal *i*, and the background value of Shaanxi soil is used in this study. *RI* is the potential ecological risk index under the comprehensive influence of heavy metals.

#### 2.3.6. Selection of Evaluation Method

In recent years, the Nemerow index method, the geo-accumulation index method, and the potential ecological risk index method have been widely used in assessing soil heavy metal pollution levels and risk. The three assessment methods have their advantages and disadvantages. Among them, the Nemerow index method overemphasizes the impact of heavy metal elements with the most extensive pollution index on environmental quality, which may artificially exaggerate or reduce the effect of some elements in the evaluation. As a result, it is difficult to distinguish the difference in the soil environmental pollution degree in the calculation results [11]. The potential ecological risk index method combines environmental assessment with toxicology. It comprehensively considers factors such as multi-element synergy, toxicity level, pollution concentration, and environmental sensitivity to heavy metal pollution. However, due to the combined effects of multiple factors, the direct use of Hakanson’s classification standard will lead to a poor accuracy of results. When studying the quantitative index of the heavy metal pollution degree in soil, the geological accumulation index method comprehensively considers the lithology, maximum value, toxicity variance of different heavy metals, and the comprehensive impact of compound pollutants. This reflects the natural distribution characteristics of heavy metals and discriminates the effects of human activities on the environment to a certain extent [12]. In this study, it was found that the content of Hg exceeded the risk screening value of agricultural land, and other elements were also enriched to different degrees. After careful consideration, the geological accumulation index method was finally selected to evaluate soil heavy metal pollution.

## 3. Results and Analysis

### 3.1. Heavy Metal Content Analysis

**Figure 3:**
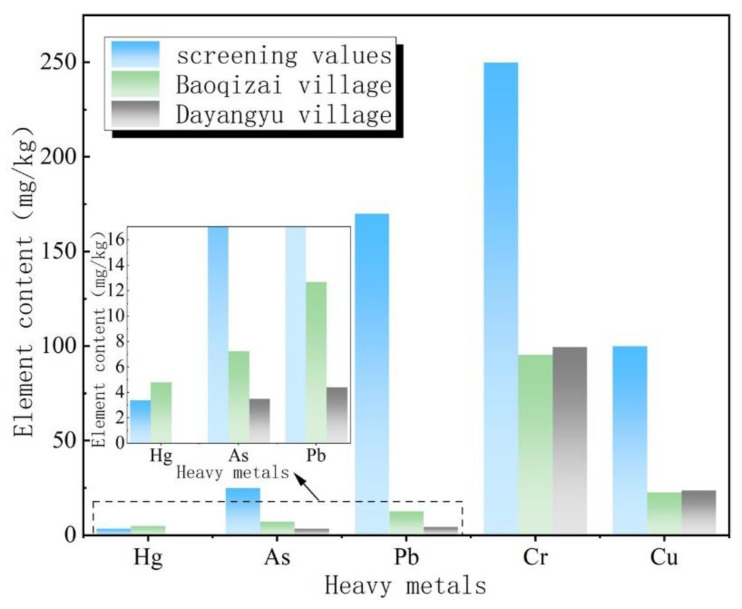
Comparison of the heavy metal content in the sample area with the screening value of agricultural land.

Table 4 shows the background values of heavy metals in the soil samples of Baoqizhai Village and Dayangyu Village tested by ICP. The pH of the soils in Baoqizhai Village and Dayangyu Village was between 6.5 and 7.5. It could be seen that the content of Fe and Mn in the soil samples of Baoqizhai and Dayangyu was relatively high, of which the content of Fe reached up to 48,550.00 and 54,789.89 mg/kg, respectively, and the content of Mn was 880.00 and 861.47 mg/kg, respectively. In addition, the Hg content in soil samples of Baoqizhai Village was 4.80 mg/kg, which was higher than the risk screening value of the “Soil Environmental Quality Risk Control Standard for Soil Contamination of Agricultural Land” (GB15618-2018) (Hg = 2.4 mg/kg). By comparing the average content of heavy metals in the two sample areas with the risk screening value of agricultural land soil (Figure 3), Hg is the heavy metal that causes severe soil pollution in Baoqizhai Village, and the heavy metals in the soil of Yangyangyu do not exceed the standard. In general, the heavy metal pollution in the soil of Baoqizhai Village is more severe than that in Dayangyu.

### 3.2. Evaluation of Heavy Metal Pollution Characteristics

To further explore the accumulation of heavy metals in the study area, the soil background value in Shaanxi Province was selected as the evaluation standard to calculate the accumulation index of heavy metals in soil. The cumulative index evaluation results (Table 5) showed that the cumulative index of Hg in Baoqizhai Village was 3.42, an extensive pollution degree. Therefore, the accumulation of Hg was worthy of attention. On the other hand, Cr was at a none-to-moderate pollution degree, and Hg, Cr, and Cu in the soil exceeded the soil background values in Shaanxi Province. The cumulative pollution of Cr in Yangyangyu Village was the most serious, reaching light pollution, and both Cr and Cu elements exceeded the soil background values in Shaanxi Province. The cumulative index intensity of five heavy metals in Baoqizhai Village was Hg > Cr > Cu > As > Pb, and the cumulative index intensity of four heavy metals in Dayangyu Village was Cr > Cu > As > Pb.

The field investigation shows that mining production is the leading cause of Hg pollution. The mining, smelting, and use of mineral resources will produce a large amount of mercury-containing waste residue, wastewater, and dust, resulting in surface water and soil pollution. In addition, mercury-based pesticides and fertilizers can bring Hg into the soil. Furthermore, industrial wastewater and domestic sewage containing trace Cr and other heavy metal elements are used to irrigate farmland, and long-term irrigation causes the enrichment of heavy metals in soil. As Baoqizhai Village and Dayangyu Village show a slight Cr enrichment phenomenon, it is speculated that it is greatly affected by the soil parent material.

### 3.3. Trend of Soil Heavy Metal Concentration in the Study Area

**Figure 4:**
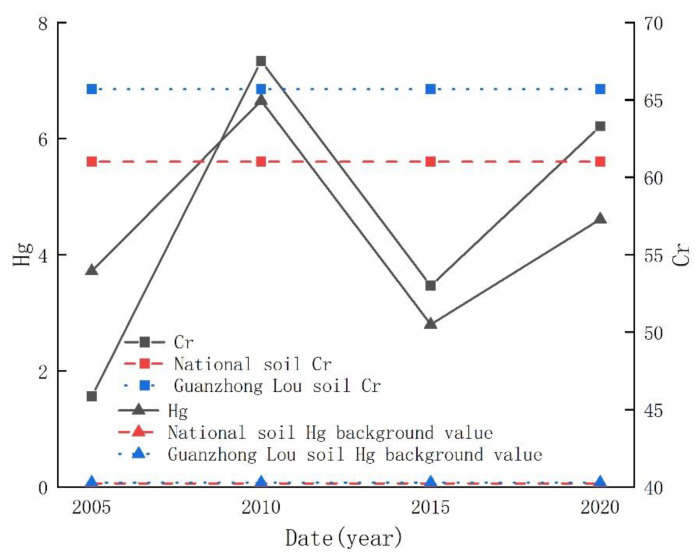
Comparison of average contents of Hg and Cr in soil in different years.

In the past, some scholars investigated the heavy metal pollution of farmland soil around the Qinling Mountains. Although the study area is not entirely consistent with this paper, comparing research results in different years is conducive to the change of soil heavy metal content in the study area over time. According to the research on the heavy metal content of farmland soil around the Qinling Mountains by several scholars in 2005, 2010, 2015, and 2020 [13,14], the average content change trend of Hg and Cr in different years was drawn, as shown in Figure 4.

It can be seen from Figure 4 that the average contents of Hg and Cr in the soil in 2020 were significantly higher than those in 2005 and 2015, while the concentrations of Hg and Cr in 2010 were the highest in comparison years. The average content of Hg in the study area has always far exceeded the national soil background value and the background value of Guanzhong Lou soil, so the pollution of Hg deserves attention. Furthermore, compared with 2005, the concentration of heavy metals in soil has increased, indicating that the pollution of farmland soil in the study area caused by human activities has increased over time.

## 4. Discussion

With the rapid development of industry and agriculture, soil heavy metal pollution has become a significant problem in most countries. More than half of the global contaminated soil plots are polluted by heavy metals such as Cd, Pb, Cu, Zn, Hg, Cr, As, Mn, Ni, and Se, among which Cd, Cu, Pb, Cr, and Hg are the primary heavy metal pollutants. Currently, the main sources of soil heavy metal pollution in the world include atmospheric deposition, sewage irrigation, industrial and mining activities, agricultural production activities, improper stacking of solid waste, etc. Studies have shown that Zn pollutes UK farmland soil, Cu, Ni, Pb, Cd, Cr, As, and Hg, and atmospheric sedimentation is the primary source of heavy metals input, accounting for 25~85% [15].

Since there is no significant difference in hydrometeorological factors around the study area, the characteristics of aquifers are consistent, and the characteristics of pollution sources are relatively fixed, the investigation of the pollution sources in the surrounding areas has great reference value for the study area. By studying the correlation between heavy metals in soil samples, it was found that the correlation coefficient between Cr and Hg was only 0.153, which inferred that Hg and Cr came from different pollution sources [16]. The research on heavy metal pollution of farmland soil in a mining area of the Qinling Mountains showed that [17], in the upper, middle, and lower layers of soil at the same site, the content of Cr was almost unchanged. The change rate of surface and deep layers was only 5.8%, indicating that Cr was more affected by soil formation. However, Hg in the surface layer was obviously higher than in the middle and deep layers, and the change rate was as high as 85.7%. The content of Hg showed a downward trend with the depth increase, indicating that Hg was affected by human activities. Related studies [18] have shown that Hg originates mainly from industrial and mining activities, atmospheric deposition, and a local superimposed contribution of high mercury pesticide. Cr is controlled primarily by the soil parent material, and a few superimposed contributions of industrial and agricultural activities, which is consistent with the results of this study.

### 4.1. Atmospheric Deposition

In many industry developed countries, the contribution of atmospheric sedimentation to the accumulation of heavy metals in the soil system ranks first among various exogenous input factors. In the total input of heavy metals, such as As, Cr, Hg, and Pb in farmland soil, the contribution rate of atmospheric deposition accounts for 43–85% [19]. Atmospheric sedimentation can be divided into dry and wet sedimentation. Heavy metals can enter the atmosphere through fossil fuel combustion, automobile exhaust, industrial flue gas, dust, mining, automobile tire wear, etc. They can be adsorbed on aerosols and then enter the soil through dry and wet sedimentation. Although they accumulate in the topsoil to varying degrees, the level of elements emitted by human activities is much higher than the natural background value. The correlation analysis between dry and wet sedimentation and soil showed that about 50% Pb and 72% Hg in paddy soil in the Suzhou area were derived from atmospheric sedimentation [20]. In Britain, 50% of farmland soil cadmium pollution comes from atmospheric sedimentation, and nearly 45% of cadmium enters the atmosphere by zinc ore smelting [21].

Due to the mining and smelting, large-scale vehicle transportation, and industrial production around the study area, a large number of harmful gases and dust containing heavy metals will be produced. After entering the atmosphere, they can spread to areas several kilometers away from the emission source and then enter the soil through dry and wet deposition, resulting in soil heavy metal pollution. This also led to atmospheric deposition becoming the primary source of soil heavy metal pollution in the northern foot of the Qinling Mountains and even the Guanzhong area.

### 4.2. Sewage Irrigation

Sewage irrigation is an important measure to promote the recycling of sewage. However, most of the irrigated sewage is untreated, resulting in many toxic and harmful substances in the sewage. For example, industrial sewage and aquaculture sewage often contain a high amount of heavy metals. Long-term irrigation will quickly lead to the accumulation of heavy metals in the soil. China is a large agricultural country. Agricultural water consumption accounts for more than 60% of the total water consumption in China, and the annual water shortage in agricultural production can reach 3 × 10^10^ m^3^ [22]. The research shows significant differences in the accumulation of soil nutrients between sewage and water irrigation, and there are different degrees of heavy metal accumulation [23]. The soil heavy metal pollution caused by sewage irrigation is increasing in some areas of China [24]. In the Xi’an sewage irrigation area, long-term sewage irrigation produced significant heavy metal accumulation in the soil [25].

### 4.3. Agricultural Production Activities

Unreasonable agricultural production activities also easily lead to heavy metal pollution in soil. In agricultural production, the excessive use of pesticides and fertilizers containing Pb, Hg, Cd, Zn, etc., will lead to the pollution of heavy metals in the soil. During the growth of crops, these substances will be absorbed and concentrated in agricultural objects, thus causing harm to human health. In livestock breeding, the increase in Cu, Zn, Pb, Cd, Hg, and other elements in feed additives leads to the more common situation that the heavy metals in livestock excreta exceed the standard. If the excrement is piled up without treatment, it will enter the soil from the surface through natural sedimentation and rain shower to cause pollution [26].

## 5. Conclusions

(1) The farmland soil at the northern foot of the Qinling Mountains was seriously polluted by Hg and Cr. The contents of Cr in Baoqizhai Village and Dayangyu Village exceeded the soil background values in Shaanxi Province. Moreover, Hg in Baoqizhai Village (4.8 mg/kg) exceeded the screening value standard of soil pollution risk in other agricultural lands (2.4 mg/kg, 6.5 < pH ≤ 7.5). So, there is a particular cumulative risk.
(2) The results of cumulative index evaluation showed that the cumulative index of Hg in farmland soil in the study area was 3.42, reaching the degree of heavy pollution, and the Cr element in the two sample areas had reached mild pollution. The cumulative index intensity of five heavy metals in Baoqizhai Village was Hg > Cr > Cu > As > Pb, and the cumulative index intensity of four heavy metals in Dayangyu Village was Cr > Cu > As > Pb.
(3) According to the investigation and analysis of the sources of heavy metals in farmland soil at the northern foot of the Qinling Mountains, atmospheric sedimentation is the primary source of heavy metal pollution in soil. Sewage irrigation and agricultural production activities also make contributions. The excessive Hg in the soil is mainly related to the waste gas, wastewater, and residue generated in industrial and mining activities. The unreasonable use of chemical fertilizers, pesticides, etc., is also one of the essential factors for excessive Hg. Cr pollution is greatly affected by regional soil parent material.

This paper analyzes the characteristics and input pathways of heavy metal pollution in agricultural land at the northern foot of the Qinling Mountains. The primary pollution sources and relative contributions were analyzed to control the soil heavy metals source better. It provides a scientific basis for the prevention and remediation of heavy metal pollution in regional and global soil. Furthermore, it provides experience for exploring a replicable and popularized heavy metal ecological risk assessment.

## Figures and Tables

**Table 1:** Soil pollution risk screening value of agricultural land [7].

Pollutant Item		Hg		As		Pb		Cr		Cu	
		Paddy Field	Other	Paddy Field	Other	Paddy Field	Other	Paddy Field	Other	Orchard	Other
Risk screening value	pH ≤ 5.5	0.5	1.3	30	40	80	70	250	150	150	50
	5.5 < pH ≤ 6.5	0.5	1.8	30	40	100	90	250	150	150	50
	6.5 < pH ≤ 7.5	0.6	2.4	25	30	140	120	300	200	200	100
	pH > 7.5	1.0	3.4	20	25	240	170	350	250	200	100

**Table 2:** Geo-accumulation index and classification [10].

*I_geo_*	≤0	(0,1]	(1,2]	(2,3]	(3,4]	(4,5]	>5
Pollution degree	None	None to moderate	Moderate	Moderate to extensive	Extensive	Extensive to extreme	Extreme

**Table 3:** Environmental background values of heavy metals in Shaanxi Province [9].

Heavy Metal	Cr	Pb	Cu	As	Hg
Soil background value	62.5	21.4	21.4	11.1	0.3

**Table 4:** Background values of heavy metals in soil samples from Baoqizhai Village and Dayangyu Village (ICP test).

Element (mg/kg)	Fe	Mn	Zn	Cr	Cu	Pb	As	Hg	Cd	Sb
Baoqizhai	48,550.00	880.00	99.50	95.50	22.60	12.70	7.25	4.80	-	-
Dayangyu	54,789.89	861.47	165.24	99.47	23.72	4.40	3.50	-	-	-

**Table 5:** Assessment results of heavy metal pollution index.

Evaluation Index	Baoqizhai					Dayangyu				
	Hg	As	Pb	Cr	Cu	Hg	As	Pb	Cr	Cu
Single factor pollution	1.41	0.29	0.074	0.38	0.23	-	0.14	0.026	0.40	0.24
Comprehensive ecological risk	1.054					0.31				
Geo-accumulation	3.42	−1.20	−1.34	0.027	−0.51	-	−2.25	−2.87	0.085	−0.44
Single factor potential ecological risk	640	6.53	2.97	3.06	5.28	-	3.15	1.028	3.18	5.54
Potential ecological risk	657.84					12.91				

## Data Availability

Not applicable.
